# The MRL Mitochondrial Genome Decreases Murine Muscular Dystrophy Severity

**DOI:** 10.3390/muscles2010005

**Published:** 2023-01-16

**Authors:** Jenan Holley-Cuthrell, Aqsa Iqbal, Ahlke Heydemann

**Affiliations:** 1 The Department of Physiology and Biophysics, The University of Illinois at Chicago, Chicago, IL 60612, USA; 2 The Center for Cardiovascular Research, Chicago, IL 60612, USA

**Keywords:** muscular dystrophy, genetic modifiers, mitochondrial genomics, MRL mouse strain

## Abstract

It is well known that muscular dystrophy disease severity is controlled by genetic modifiers. The expectation is that by identifying these modifiers, we can illuminate additional therapeutic targets with which to combat the disease. To this end we have been investigating the MRL mouse strain, which is highly resistant to muscular dystrophy-mediated fibrosis. The MRL mouse strain contains two mitochondrial-encoded, naturally occurring heteroplasmies: T3900C in tRNA-Met, and variable adenine insertions at 9821 in tRNA-Arg. Heteroplasmies are mitochondrial mutations that are variably present in a cell’s mitochondria. Therefore, MRL cells can contain 0 to 100% of each mitochondrial mutation. We have chosen the severely affected ϒ-sarcoglycan (Sgcg–/–) deficient mice on the DBA2/J background as our muscular dystrophy model to demonstrate the effects of these mitochondrial heteroplasmies on disease severity. Mice from the (Sgcg–/–) DBA2/J (D) and wildtype MRL (M) strains were crossed for more than 10 generations to establish two separate, pure breeding mouse lines: Sgcg+/–Nuc^D^Mito^%M^ and Sgcg+/–Nuc^M^Mito^%M^. The Sgcg–/– mice from these separate lines were analyzed at 8 weeks old for membrane permeability, hydroxyproline content, pAMPK content, fibronectin content, and percentage of each heteroplasmy. We have identified that the MRL mitochondrial mutation T3900C confers a portion of the fibrosis resistance identified in the MRL mouse strain. These results have been extended to significantly correlate increased MRL mitochondria with increased pAMPK and decreased muscular dystrophy fibrosis. The beneficial mechanisms controlled by the MRL mitochondria will be discussed. We are establishing metabolic aspects of muscular dystrophy pathogenesis. These metabolic pathways will now be investigated for therapeutic targets.

## 1. Introduction

Duchenne muscular dystrophy (DMD) is an X-linked disease characterized by the absence of fully functioning dystrophin protein. The limb-girdle muscular dystrophies (LGMD) are a group of genetic diseases similar in pathology to DMD but are caused by mutations in autosomal genes. LGMD type 2C has been modelled in mice lacking ϒ-sarcoglycan by removing the first coding exon of the Sgcg gene [1]. Sgcg–/– mice develop symptoms of muscular dystrophy, including leaky plasma membranes, and increased muscle fibrosis [1]. Through breeding, the Sgcg–/– allele was introduced into the DBA/2J (Sgcg–/–D2) mouse model, which resulted in a more severe phenotype [2]. The Sgcg–/–D2 were then bred into the super-healing Murphy Roth Large (MRL) genome to determine if the pace of muscular degeneration or regeneration were altered. The resultant F2 Sgcg–/– D2/MRL mixed nuclear genome resulted in high, mutant levels of membrane permeability but reduced fibrosis in the heart, diaphragm, and skeletal muscles [2], thus, indicating that regeneration was enhanced in the MRL mice. Genome wide association studies of these F2 mice identified a novel significant peak on chromosome 2 and reaffirmed the importance of the LTBP4 polymorphism on chromosome 7 [3,4]. However, the causative polymorphism on chromosome 2 has yet to been identified.

The super healing ability of the wild-type Murphy Roth Large (MRL) mouse has been investigated by subjecting the mice to various wound and disease models (reviewed in [5]). The super healing ability of the MRL strain was discovered by its ability to quickly heal ear holes made for mouse identification [6,7]. This regenerative healing of the mouse ear wounds is characterized by reestablishment of the skin, hair follicles, nerve fibers, glands, muscle, and cartilage by 8 weeks after wounding [6]. The MRL healing ability is witnessed in a variety of situations, including digit wounding, corneal scarring, reduced scar formation upon cardiac freeze injury, coronary artery ligation, and muscular dystrophy [5,8,9,10,11], however, this ability is not seen in all wound models, and there are variable responses to the same wound model. Many mechanisms have been proposed to explain the healing phenomenon and these mechanisms have been supported by physiologic, molecular, and genetic data (reviewed in [5,12,13]).

Affected muscular dystrophy (MD) patients often exhibit defective mitochondria, and reduced energy production [14,15,16]. The body removes damaged mitochondria through autophagy or more specifically, mitophagy. Preclinical studies have shown that increasing mitophagy benefits the muscular dystrophy (mdx) mouse [17]. In this manuscript, the targeted metabolic pathway utilized AICAR, a synthetic AMP-activated protein kinase (AMPK) activator [18]. Activation is by phosphorylation of AMPK, making pAMPK, a metabolic sensor and activator of the mitophagy pathway. Chronic AICAR treatment also limits formation of the mitochondrial membrane permeability transition pore (PTP) complex, a structure that promotes swelling and release of proapoptotic factors [17]. It has also been demonstrated that pAMPK can enhance skeletal muscle regeneration [19]. Furthermore, pAMPK reduces reactive oxygen species [20,21], which are in excess in MD [22]. Therefore, there are many pieces of data that indicate the increases, natural or exogenous, in pAMPK levels benefit MD muscles, including muscular dystrophy in the MRL strain (for full reviews see [5,23]). Previously, we had shown that the wildtype MRL mice had increased levels of pAMPK in their skeletal muscles, including quadriceps, triceps brachial, and gluteal, compared to C57Bl/6 (B6) [24]. Therefore, we pursued this pathway in the current study. Not surprisingly, the MRL mouse strain also exhibits metabolic differences when compared to C57BL/6J mice due to altered mitochondrial function [24,25]. In addition, MRL mice fed a high-fat diet (HFD) did not show signs of hyperglycemia, hyperinsulinemia, insulin resistance, or hypersensitivity to glucose when compared to HFD-B6 mice [26,27,28]. The HFD-MRL mice also show heightened levels of pAMPK, suggesting a proximal mechanism for the beneficial metabolic differences, beneficial for HFD and MD pathologies [24].

The mitochondrial genome of the MRL mouse reveals heteroplasmies that differentiate it from most other mouse lines, including C57BL/6J, 129 mice [29], and DBA2/J mice (unpublished observations; A.H.). The MRL mouse strain has a synonymous variation in the DNA encoding the NADH dehydrogenase 3 protein (*mt-ND3*) as well as two non-synonymous heteroplasmies. The substitution in *ND3* was found to be inconsequential and converts an ATT initiator codon to an ATC initiator codon. One consequential heteroplasmy is a T3900C substitution in the TΨC loop of tRNA methionine gene (*tRNA-Met*; *mt-TM*), the mutant MRL nucleotide is cytosine. The other consequential heteroplasmy is the insertion of a variable number of adenine nucleotides in an adenine-tract at 9821 of the tRNA arginine gene of the MRL mice (*tRNA-Arg*; *mt-TR*). The wild-type mitochondria have 9 adenines in this tract, whereas the MRL mitochondria can have up to 13 adenines. MRL cells can therefore contain many different mitochondrial genomes. Expansion of the adenine-tract in the tRNA-Arg may lead to phenotypes such as age-related hearing loss [30]. The increased adenine-tract also increases the length of the single-stranded DHU loop but does not change the 3 A-U hydrogen bonded base pairs. This may lead to an altered secondary structure when forming a tRNA with an aminoacyl-tRNA synthetase complex and a tRNA with a ribosome complex. The T3900C heteroplasmy in tRNA-Met effects the T-arm structure of the molecule, however, until this manuscript, this mutation has not been shown as a phenotype altering mutation.

Mutations in mitochondrial DNA (mtDNA) and the subsequent protein products have been implicated in many human diseases. With little protection from histones or membranes, and decreased DNA repair mechanisms compared to nuclear genomes, more than 250 pathogenic mtDNA mutations have been discovered [31]. Utilization of both nuclear and mitochondrial genomes to encode oxidative phosphorylation protein complexes leads to issues if mtDNA mutations are prevalent. The most common mtDNA mutations result in mitochondrial encephalopathy, lactic acidosis, and stroke-like episodes (MELAS), described as a point mutation A3243G in the *tRNA-Leu* gene [32]. Furthermore, mitochondrial tRNA gene mutations are responsible for a wide variety of human diseases [33].

Utilizing specific breeding strategies in ϒ-sarcoglycan (Sgcg–/–) knockout mice, we have investigated the disease modifying impact of the two consequential MRL mitochondrial heteroplasmies. The muscular dystrophy Sgcg–/– mice were analyzed at 8 weeks of age for biochemical indicators of pathology and percentage of each mitochondrial heteroplasmy. We have identified that increased MRL mitochondrial content correlates directly and significantly with quadriceps pAMPK content and inversely with severity of disease, quantified by fibrosis. These investigations identify metabolism modulators as novel therapeutic targets for MD treatments.

## 2. Results

### 2.1. Non-Random Mitochondrial Inheritance

**Figure 1:**
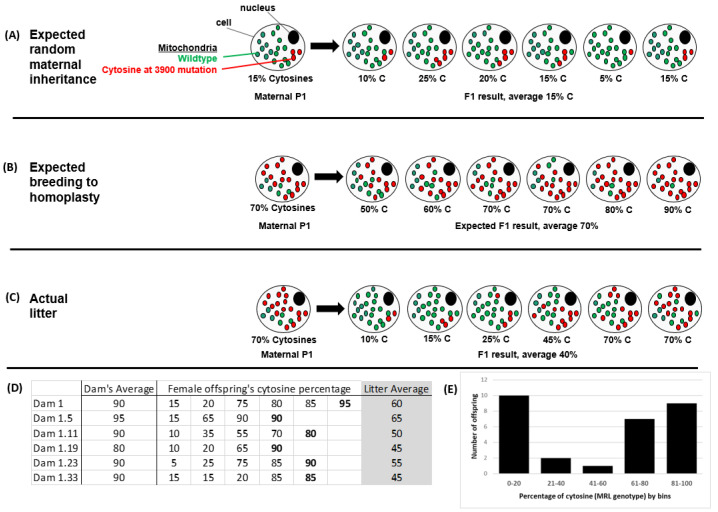
Introduction to heteroplasmies. The nature of mitochondrial segregation during oogenesis is not random in the MRL mouse strain. (**A**) The expected random segregation is illustrated, where the offspring’s percentage of mutant mitochondria would center around the dam’s percentage. (**B**) Using this randomness, we should be able to generate mitochondrial homoplasmy; i.e., mitochondrially pure breeding mouse lines. (**C**) Instead, we noted that mitochondrial genome appeared to be reset during each generation centering on ~40% mutant cytosines at the 3900 position. An actual litter is depicted. (**D**) Our breeding scheme to achieve a pure MRL mitochondrial line of mice. In each case the female offspring with the highest percentage cytosines was chosen to be the Dam (bolded) for the next generation. We, however, never got to 100% MRL and the subsequent litters consistently reset to lower values, (as seen in the average column, shaded grey). (**E**) Graphical representation of the Dam 1 offspring data, illustrating the often-noted bell-shaped nature of this mitochondrial reset.

Originally, the MRL mice were bred in an attempt to generate dams and subsequent generations containing 100% mutant (MRL) mitochondrial DNA. The attempt was first conducted for the T3900C heteroplasmy. It was expected that the heteroplasmy would segregate randomly, averaging around the percentage of the mother (Figure 1A). We therefore choose three dams with the highest percentage of cytosines and expected them to have daughters with high percentages that could be bred on (Figure 1B). However, after six generations of choosing the daughters (from the three original dams) with the highest percentage cytosine at 3900, a pure breeding line could not be established. The offspring always displayed a range of mitochondrial mutations. For example, a dam with 70% MRL mitochondria (at the T3900C site) produced a litter of 6 mice with an average of 40% MRL mitochondria (Figure 1C), homoplasticity was never achieved (Figure 1D). Furthermore, it was noticed that the offspring favored the ends of the mutation range (Figure 1E). This graph was generated from the Dam #1 pedigree. This dumb-bell shaped curve was seen in all of the litters that we analyzed. We only assessed the tail for mitochondrial DNA mutations. We did not attempt to generate a pure breeding line for the adenine expansion.

### 2.2. The MRL Mitochondrial Genome Contributes to the Muscular Dystrophy Super-Healing Phenotype

**Figure 2:**
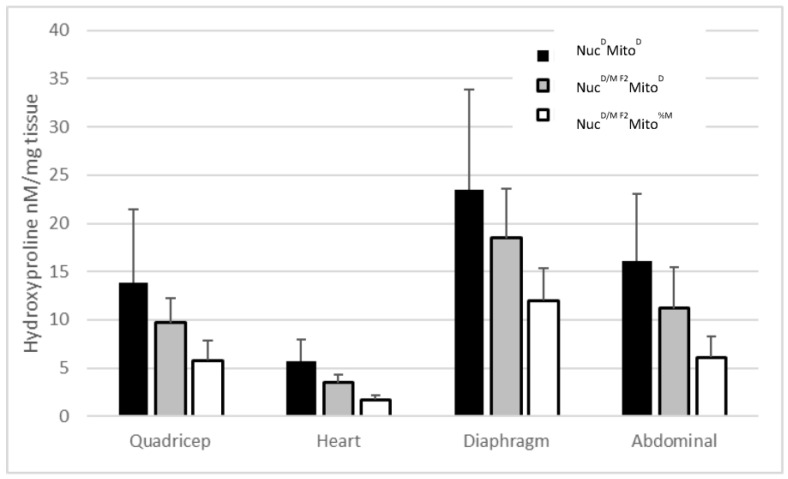
Both the MRL nuclear and mitochondrial genomes contribute to the muscular dystrophy phenotype reduction. These data are a reanalysis of the F2 mice from a previous work [4]. We used the hydroxyproline assay to measure fibrosis of the striated muscles. The Sgcg–/–Nuc^D^Mito^D^ mice have the highest hydroxyproline levels for the tissues (black bars), the F2 mice that were 50% MRL and 50% D2 nuclear with D2 maternal inheritance had some benefit of fibrosis reduction (grey bars), and the F2 mice that had 50% MRL and 50% D2 nuclear with MRL maternal inheritance had the lowest fibrosis levels (white bars). This analysis was simply based upon maternal inheritance; these mitochondrial genomes are therefore undefined heteroplasmic Mito^%M^. N > 10, averages with standard deviations.

In a proof of concept set of experiments, it was demonstrated that the ϒ-sarcoglycan mutation-mediated muscular dystrophy (MD, Sgcg–/–) F2 mice from a wtMRL × Sgcg–/– DBA2/J (D2) cross were resistant to fibrosis [4]. After the mitochondrial heteroplasmy manuscript was published by the Heber-Katz group [29], the original mouse pedigrees were reanalyzed. The original pedigrees were set up between wtMRL mice and Sgcg–/–D2 mice, which had previously been shown to have severe muscular dystrophy [2,3]. In the original manuscript, the mice were breed for two generations and the homozygous null F2 offspring were assessed for their MD phenotypes [4]. These F2 mice had high levels of membrane permeability, matching the levels identified in the Sgcg–/–D2 pure bred animals, but significantly reduced fibrosis. When reanalyzing the pedigrees, it was determined that the F2 offspring (with a nuclear genome of 50% D2 and 50% MRL) that contained MRL mitochondria—their mothers and grandmothers were MRL strain—had the lowest levels of fibrosis (Figure 2). The low levels were significantly below the parental Sgcg–/–D2 levels and the F2 mice that contained D2 mitochondria. The MRL mitochondria reduced fibrosis in all tissues analyzed: quadriceps, heart ventricles, diaphragm, and abdominals (Figure 2). This analysis was simply based upon maternally inheritance; these mitochondrial genomes are therefore undefined heteroplasmic—Mito^%M^.

**Figure 3:**
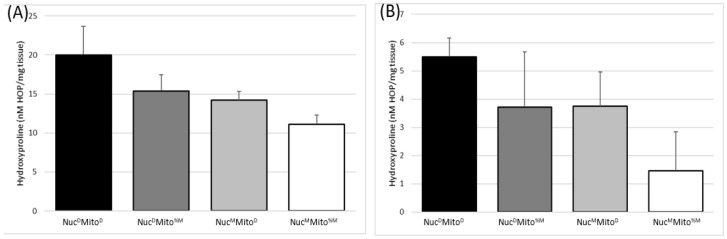
Quantitative MD phenotype analysis in the four mixed genome mouse lines. As in Figure 2 the mitochondrial genome is purely based upon maternal inheritance and the MRL mitochondrial genomes are undefined heteroplasmic (Mito^%M^). Both MRL nuclear and mitochondrial genomes decrease the fibrosis/hydroxyproline levels in the (**A**) diaphragm and (**B**) cardiac ventricles. Nuc^D^Mito^D^ (black bars), Nuc^D^Mito^%M^ (dark grey), Nuc^M^Mito^D^ (light gray), and Nuc^M^Mito^%M^ (white bars). N > 7, averages with standard deviations.

To further analyze the beneficial genetics from the MRL line, four mouse lines were generated by backcrossing for at least 10 generations. These lines separated the nuclear (Nuc) and mitochondrial (Mito) genomes of the MRL (M) and DBA2/J (D) mice: Nuc^M^Mito^%M^, Nuc^M^Mito^D^, Nuc^D^Mito^%M^, and Nuc^D^Mito^D^. These lines carried the mutant Sgcg allele as a heterozygote, and during breeding and after 10 generations, the null mice were assessed for their MD phenotypes. Corroborating the preliminary data of the F2 pedigrees (Figure 2), these four breeding lines confirmed that both the nuclear and the mitochondrial genomes of the MRL mice contributed to their enhanced healing against the MD fibrosis phenotype (Figure 3). The MRL mitochondria and nuclear genomes reduced fibrosis in diaphragm, and cardiac ventricles (Figure 3A,B).

Membrane permeability of the animals from these four lines is not significantly different (Supplementry Figure S1). Evans blue dye (EBD) uptake was used as a surrogate for membrane permeability. A difference was not expected because the original Sgcg–/–MRL × D2 F2 mice did not display a difference in EBD uptake when compared to the Sgcg–/–D2 mice [4].

To this point we have investigated the nuclear genomes with Mendelian inheritance and mitochondrial genomes with maternal inheritance; so far, no regard has been made to the percentage of mitochondrial genome i.e., heteroplasmy, in the individual animals.

### 2.3. Results of Mitotyping Analysis

After it became clear that the MRL mitochondria reduces fibrosis in mice with gamma ϒ-sarcoglycan mediated MD, more mitochondrially precise investigations were conducted. Because the MRL mitochondrial heteroplasmies could not be bred to purity, we took advantage of the heteroplasmies and quantified the percentage of MRL mutant polymorphisms in each animal. In this manner, the dose-response effect of each heteroplasmy could be investigated as we correlated percent heteroplasmy with fibrosis.

**Figure 4:**
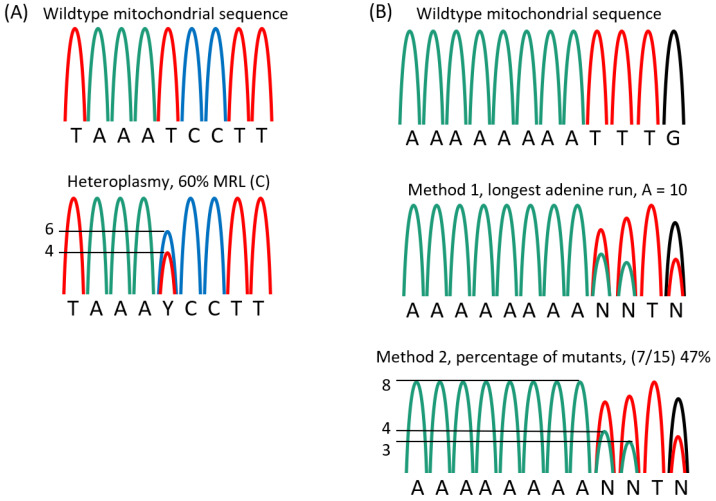
Mitotyping analysis. (**A**) Percent cytosine at position 3900 was measured from the nucleotide peaks of a standard sequencing application. (**B**) Adenine length was assessed as the last visible adenine peak or by measuring the height of the normal, ninth adenine, assigning this as 100% and comparing this to the sum of the longer adenine peaks ((ΣA_9–15_)/A_8_) × 100).

The mitotyping analysis of the tail tissue allowed for quick and accurate assessment of the heteroplasmies in the mice with MRL mitochondria. For the T3900C polymorphism, the height of the cytosine peak was divided by the sum of the heights from both C and T peaks and multiplied by 100 (Figure 4A), thus, enabling us to report the percentage of mitochondria genomes that have a cytosine at 3900. For the adenine insertions the quantification procedure was altered, the normal number of adenines was 9 and the mutants had 10 or more adenines, up to 13 [29]; a simple percentage would not reflect the full story. Therefore, multiple quantification methods were utilized: 1, the longest run of adenines was used to quantify the mutation levels (Figure 4B), and 2, the percentage of ten or more adenines was calculated for percentage of heteroplasmy. Neither of these methods reflected the possible different effects of different adenine tract lengths, thus we are naively assuming a mutation is a mutation. Future biochemical analyzes must be done to investigate the functional differences of adenine tract length and the T3900C heteroplasmy.

### 2.4. The Two Mitochondrial Heteroplasmies Differentially Affect MD Fibrosis Pathology

The two non-synonymous mitochondrial heteroplasmies were quantified in the mice that contained MRL mitochondria, based upon their maternal inheritance patterns. Therefore, only the Sgcg–/–Nuc^D^Mito^%M^ and Sgcg–/–Nuc^M^Mito^%M^ lines were mitotyped. In these two strains it was identified that the T3900C mutation inversely segregated with fibrosis, while the A-tract insertion (by either method of quantification) did not affect fibrosis levels.

**Figure 5:**
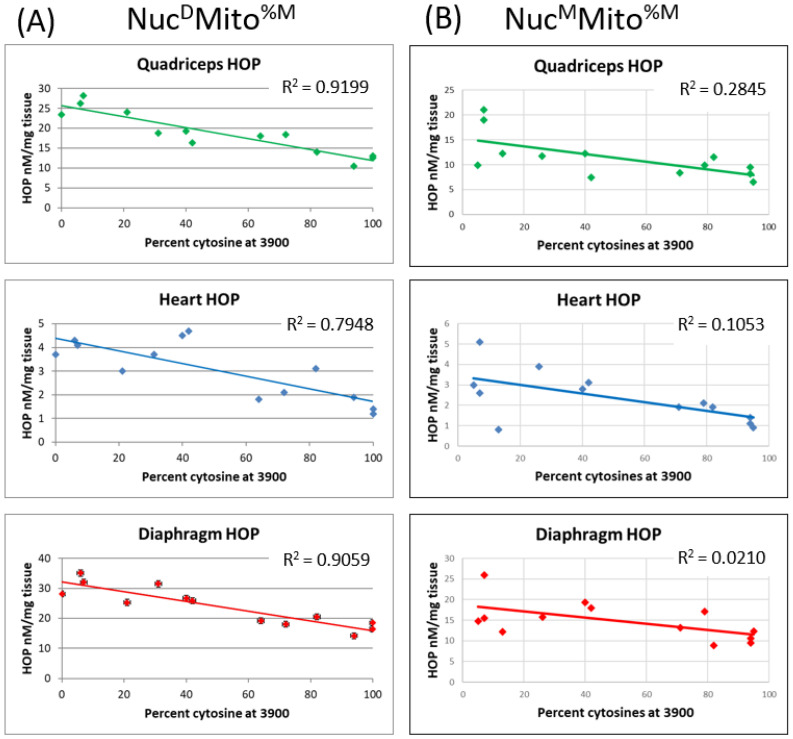
The cytosine mutation at nucleotide 3900 segregates with decreased fibrosis. The percentage of MRL cytosine at position 3900 correlates inversely with fibrosis, in quadriceps, diaphragm and cardiac ventricles: *p* < 0.05 for all three tissues. (**A**) This is true in the mice with D2 nuclear genomes and (**B**) mice with MRL nuclear genomes.

In all three muscle tissues analyzed (the diaphragm, quadriceps, and heart ventricles), the T3900C mutation levels significantly correlated with reduced fibrosis in the Sgcg–/–Nuc^D^Mito^%M^ line (Figure 5A). In each of these tissues, the more MRL mitochondria present (the percentage of cytosines at nucleotide 3900), the lower the fibrosis. The Pearson’s coefficients for each of the tissues demonstrated a significant (*p* < 0.05) correlation between the percentage of cytosines and reduced fibrosis. When the correlation analysis was conducted for the number of adenines in the adenine tract, no significant correlations were observed in any of the muscle tissues, (*p* > 0.05, Supplement Figure S2A).

These same analyses were conducted in the Sgcg–/–Nuc^M^Mito^%M^ mouse line. This mouse line, with the MRL nuclear genome and a percentage of MRL mitochondrial genome, also demonstrated decreased fibrosis with increasing MRL T3900C mitochondrial percentage (Figure 5B). Due to the MRL nuclear genome, all of the HOP values were decreased, and additionally, we saw a further HOP decrease significantly correlated with percentage T3900C (*p* < 0.05). Again, there was no fibrosis effect from changes in the adenine tract length (Supplement Figure S2B).

This lack of correlation at the adenine tract allows a more straightforward investigation of the percentage of cytosines at the nucleotide 3900 correlation.

### 2.5. The MRL Mitochondrial C3900T Heteroplasmy also Correlates with pAMPK Levels and Inversely Correlates with Fibronectin Levels

To initiate the investigations of how the C3900T heteroplasmy is affecting the metabolism and regeneration abilities of the Sgcg–/– mice, phosphorylated, activated adenine monophosphate activated protein kinase (pAMPK) and fibronectin levels were assessed by immunoblot of the quadriceps. In the quadriceps skeletal muscles, the level of pAMPK correlates with the percentage of the MRL mitochondrial T3900C mutation.

**Figure 6:**
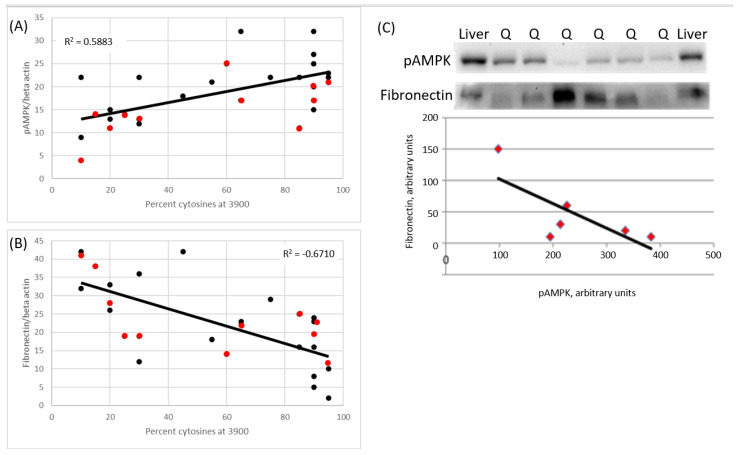
The cytosine mutation at nucleotide 3900 segregates with molecular markers of decreased fibrosis. Both lines of mice with variable MRL mitochondria show the significant (*p* < 0.05) positive correlation of the percentage of cytosines to pAMPK, normalized for beta actin (**A**), and the significant (*p* < 0.05) inverse correlation of the percentage of cytosine and fibronectin protein, normalized for beta actin (**B**). Nuc^D^Mito^%M^ in red and Nuc^M^Mito^%M^ in black. Representative blots of pAMPK and fibronectin. The center lanes are quadriceps from a single litter of Nuc^M^Mito^%M^ mice. The flanking lanes contain control liver samples used to quantify between blots. The blot was stripped between each primary antibody blot. This blot demonstrates a significant (*p* < 0.05) negative correlation between pAMPK and fibronectin protein (both normalized for beta actin) (**C**).

From the immunoblot results, we identified that the quadriceps pAMPK levels strongly correlate with the percentage of cytosines at 3900 (Figure 6A and Table 1). A total of 17 mice from the Sgcg–/–Nuc^M^Mito^%M^ line (black dots) and 11 mice from the Sgcg–/–Nuc^D^Mito^%M^ line (red dots) were analyzed for pAMPK levels and correlations to percent of cytosine at 3900 were calculated. Also shown is the best fit line, which was plotted using Excel, (version 2202).

These same samples were also analyzed for fibronectin levels. In this case the correlation was inverse: the more MRL mitochondria that the mice had, the lower their fibronectin levels (Figure 6B and Table 2).

Since we had the values before us, we were able to reconfirm our earlier data, which was that the MRL nuclear genome correlated with increased pAMPK levels. A Student’s *t*-test resulted in a significance of *p* = 0.029 for this comparison. When analyzing the MRL nuclear genome for fibronectin protein levels, the Student’s *t*-test yielded a non-significant value. Representative immunoblots for pAMPK and fibronectin from a single litter are shown (Figure 6C). In this case the inverse correlation between pAMPK and fibronectin is seen, although not statistically significant.

These same samples of pAMPK and fibronectin were assessed for correlation with the length of the adenine tract and with the percentage of mutant genomes at adenine tract. No correlations were identified with either method of quantifying the adenine length mutation.

## 3. Discussion

The MRL mouse line has long been examined for its super-healing abilities [4,5,6,14]. There are many investigations into the genetic and molecular mechanisms leading to the super-healing phenotype [4,12]. To identify the genetics causing the super-healing phenotype of the MRL mice, multiple investigators have employed genome-wide association studies [5,12]. Although many chromosomal regions have been identified as segregating with the MRL regenerative phenotype, none has resulted in identification of a gene. Too many low peaks were found, indicating multiple causative nuclear genes, each contributing partially to the phenotype and, therefore, making it difficult to identify. This left the possibility that the regenerative genetics were epigenetic. Therefore, we have been pursuing the mitochondrial genome as causative for a portion of the MRL benefits. This hypothesis has now been supported. Perhaps the original GWAS studies could be reanalyzed using the percentage of MRL mitochondria as a covariate.

This current manuscript identifies significant MD benefits provided by both the MRL nuclear and mitochondrial genomes (Figure 2 and Figure 3). This work corroborates that the MRL nuclear genome is beneficial [4]. Now we realize that the MRL mitochondrial genome also benefits the MD mice. From Figure 3, we can surmise that the benefits of the nuclear and mitochondrial genomes are approximately equal because the central two bars in each of these graphs display similar HOP levels. We have extended these results by taking advantage of the heteroplasmy nature of the MRL mitochondria. We have shown a dose-response correlation for the T3900C mutation and muscular dystrophy benefit (Figure 5).

We have also shown that the MRL mitochondrial genome correlates with an increase in pAMPK (Figure 6). Further investigations are required to identify the particular molecular pathways that lead from the MRL mitochondrial mutations to the increased pAMPK. From previously published work, it appears that AMPK protein itself is not upregulated [24,26], however, more precise and sensitive investigations, such as RT-qPCR of the AMPK family members, are in order. Additional investigations are also required to identify the interactions between the two genomes. Understanding the stoichiometry of the mitochondrial components will further the understanding of mixing the genomes.

It is interesting to note that neither the MRL nuclear nor mitochondrial genomes reduce the Evans blue dye uptake (used to quantify membrane permeability) in the F2 MRL/D2 Sgcg–/– mice [4], in the Sgcg–/–Nuc^M^Mito^M^ strain, or the Sgcg–/–Nuc^M^Mito^D^ strain (Supplemental Figure S1). These various mouse strains do get the early disease marker phenotype of membrane permeability at high Sgcg–/– DBA2/J levels. This argues that the MRL mice are truly super-healing and can regenerate their skeletal muscles from a quantified severe phenotype.

The other MRL heteroplasmy—adenine-tract insertion—may segregate with regeneration if different phenotypes were assessed, however, the adenine tract length did not correlate to fibrosis (Supplementary Figure S2). It may also be that the adenines should be counted in a different manner, such as the percentage of 9 adenines or the wild type mitochondria, or that one specific mutant length might be most beneficial. These experiments remain to be conducted.

Although this study was not designed to investigate heteroplasmic mitochondrial inheritance patterns, some interesting observations were made. For instance, we noted that breeding to homoplastic mitochondria was not achievable in the 5 or 6 generations for which we attempted this. We never achieved the favorable “bottleneck” that would produce homoplastic mitochondria. This data is in contrast to genetically engineered heteroplasmic cells containing NZB and 129S6 mitochondria on a C57BL/6J nuclear background [34]. The simple answer may be that some heteroplasmies are detrimental, such as in the Sharpley manuscript, which identified increasing homoplasticity, and some heteroplasmies are relatively neutral, such as in the mice of this manuscript. The MRL mitochondria lessen the MD disease burden, but likely do little for the overall health and fecundity of the mice. Therefore, the segregation pattern may follow a simple survival of the fittest mitochondria. We are unsure of where the reset occurs; why a dam with high MRL cytosines produces a litter with an average of 40% MRL mitochondria. It may be during oogenesis [35] or it may be a function of different tissues containing different amounts of mutations [36].

We also noted a dumbbell shaped inheritance pattern, where the extreme abundance (>60%) or extreme lack (<30%) of the T3900C MRL mutation was favored (Figure 1E). There were very few mice with T3900C in the 30% to 60% range. Additional, properly designed experiments would need to be conducted to identify the reasons for this pattern of segregation.

This study has some important limitations to discuss. We have been careful to state that the mitochondrial T3900C heteroplasmy significantly correlates with a milder phenotype when compared to the normal murine mitochondrial genome. With the additional correlations to pAMPK and fibronectin levels, we are now hypothesizing that the heteroplasmy is the cause of the improved phenotype. Of course, additional experiments must be conducted to understand the molecular cascades of naturally increasing the pAMPK levels. Additional biochemical assays must also be conducted to understand the exact metabolic impact of each mitochondrial mutation. Another limitation of the current study is that our mitotyping was performed on tail tissue from two-week-old mice. There is significant literature that tissue type [36], age [37], and sex [38] affect mitochondrial genome and function. These affects could be changes in mutation (heteroplasmy) load, mitochondrial content per cell, mitophagy, and/or autophagy. The stability of the heteroplasmies must also be analyzed in the future.

A further limitation is that by using additional mice, we may have identified phenotypic correlations for the adenine tract data. We quantified the adenine tract length by two separate methods, and neither method demonstrated any phenotypic correlation. The same number of animals demonstrated a correlation for the T3900C correlation. Thus, we can deduce that there may be adenine tract affects, but they would be less than the T3900C.

## 4. Conclusion

This work was initiated to identify additional molecular targets for future MD therapies. Metformin, which is known to increase pAMPK levels, is in co-therapy clinical trials with L-arginine (NCT02516085) and has proven successful in metabolic changes and phenotype decreases ([39]). This current manuscript would argue for the design of next generation AMPK activators. This current work would also argue for future (and perhaps past) MRL genome wide association studies being conducted with mitochondrial content as a covariate.

## 5. Materials and Methods

### 5.1. DNA Purification and Mitotyping

Total cellular DNA was purified from a ~1mm sample of frozen mouse tail using the Puregene kit following the standard written protocol. The DNA was hydrated with 50 µL of 1 × TE. Primers m07 and m29 were used for the C/T heteroplasmy, while primers m30 and m31 were used for the adenine tract mutation (Table 3). Choice Blue Mastermix (Denville, Bath, UK; #CB4065-8) and filtered milliQ (St. Louis, MO, USA) water were used. The PCR conditions for both protocols are as follows: initial denature at 94 °C 5 min, followed by 30 cycles of 94° for 30 s, 58° for 45 s, and 72° for 45 s, with the final hold at 72° for 5 min. The resulting samples were run on a 2% agarose gel, and each of the bands of DNA were purified using the Gel/PCR DNA Fragment Extraction KIT (IBI Scientific, Dubuque, IA, USA). The excised purified DNA was sent to UIC Research and Resources Core Lab (Chicago, IL, USA) for sequencing with the appropriate primers.

The samples were analyzed for length of adenine chain in the DNA coding for the tRNA-Arg, and for the percentage of cytosine in the DNA coding for tRNA-Met. The sequenced DNA fragments were analyzed using SeqMan Pro (Madison, WI, USA). Measuring was done by manual assessment of peak height using a ruler (Figure 4). The location of the C/T mutation was found in the DNA fragment and the percent C/T was calculated by the ratio peak heights ((C/(T + C)) × 100). Results are reported as the percentage of cytosine (the mutant, MRL nucleotide, results were rounded off to the nearest 5 percent increment). The length of the adenine chain was counted by 2 different methods: 1, as the longest adenine chain visible by sequence and recorded as a whole number, and 2, by measuring the height of the normal, ninth adenine, and comparing this to the sum of the longer adenine peaks (((ΣA_10–15_)/A_9_) × 100), which results as a percentage of the longer, mutant adenines.

### 5.2. Immunoblot

Frozen quadriceps muscle tissues were prepared for pAMPK, fibronectin, and beta-actin immunoblotting, and analyzed as done previously in [24]. Briefly, the tissues were homogenized (Tissue Ruptor, Qiagen, Ann Arbor, MI, USA) at the full setting for 1 min on ice in lysis buffer (20 mmol/L HEPES [pH 7.4], 50 mmol/L b-glycerol phosphate, 2 mmol/L EGTA, 1 mmol/L DTT, 10 mmol/L sodium fluoride, 1 mmol/L sodium orthovanadate, 1% TritonX-100, 10% glycerol and protease/phosphatase inhibitors (ThermoScientific, 1862209, Waltham, MA, USA). The slurry was spun in a microfuge at 16,000 RPM for 2 min, and then the supernatants were transferred to clean tubes. The Bradford assay (Biorad, Hercules, CA, USA) identified the protein concentrations: 25 ug was denatured by boiling in Lammelli buffer and loaded per lane of a 12% TGE gel (Biorad, Hercules, CA, USA). Wet transfer to a PVDF membrane was followed by Ponceau staining, blocking in 3% bovine serum albumin in tTBS (Tris buffered saline with 1% tween 20) at room temperature for 30 min. Primary antibodies pAMPK, fibronectin, and beta-actin (Cell Signaling Technologies, Danvers, MA, USA) were diluted 1:500 in tTBS and incubated on the blot overnight at 4C with shaking. After three 15-min washes in tTBS, the appropriate secondary antibodies were incubated on the gels for one hour at room temperature. Three final 15-min washes were done before visualization with enhanced chemiluminescence (Amersham, Buckinghamshire, UK) with a Chemidoc (Biorad, Hercules, CA, USA).

### 5.3. Evan’s Blue Dye

Evan’s blue dye (EBD) assessment was done on the triceps, gastrocnemius/soleus, gluteus/hamstrings, half of the abdominals, half of the quadriceps muscles, and both kidneys for EBD injection control. The EBD solution was prepared and administered as described in [2,4]. Forty hours later, the tissues were dissected, incubated at 55 °C in 500 μL formamide for at least 2 h, and absorbance was measured at 580 nM. The results are reported in (muscle absorbance/mg tissue)/(kidney absorbance/mg tissue).

### 5.4. Hydroxyproline Assay

In order to assess fibrosis, a hydroxyproline assessment (HOP) was performed as described in [2,4]. The tissues harvested for the HOP assay were a portion of the abdominals, cardiac ventricles, diaphragm, and half of each quadriceps muscles. The tissues were incubated in 500 μL 6M HCl at 105C overnight. Ten microliters of the hydroxylate was transferred to a new microcentrifuge tube, which received 150 μL of isopropanol followed by 75 μL of chloramine T (7% solution in water) mixed 1:4 with citrate acetate buffer (1.44 M sodium acetate, 5.75 M citric acid, 0.435 M NaOH, 3.85% of isopropanol in water). The mixture was vortexed and incubated at room temperature for 10 min. Then, 1 mL of Ehrlich reagent (0.3% p-dimethylaminobenzaldehyde in ethanol with 6.75% sulfuric acid) diluted 3:13 in isopropanol was added. This mixture was vortexed and incubated for 30 min at 58 °C, the reaction was quenched on ice for 5 min before a quick centrifugation and reading the supernatant at 620 nM. A standard curve with hydroxyproline was generated. Therefore, the results are reported in nM HOP/ug tissue.

### 5.5. Statistics

Pearson’s coefficient was used to determine if a significant relationship existed between any of the resulting data. Collected data was entered into Microsoft Excel (version 2202) and the Pearson’s coefficient function was used between the data columns.

### 5.6. Ethical Approval

This study was approved by the Institutional Animal Care and Use Committee (IACUC) of the University of Illinois at Chicago, which is accredited by the American Association for the Accreditation of Laboratory Animal Care (AAALAC). All animals received humane care in compliance with the *Principles of Laboratory Animal Care* formulated by the National Society for Medical Research and the *Guide for the Care and Use of Laboratory Animal Resources* published by the US National Institutes of Health.

## Figures and Tables

**Table 1:** Correlation of pAMPK and the percentage of cytosines.

	N	df	Pearson’s Correlation	*p*<
All Sgcg–/– mice	28	26	0.5883	0.01
Sgcg–/–Nuc^M^Mito^%M^ mice	17	15	0.6006	0.05
Sgcg–/–Nuc^D^Mito^%M^ mice	11	9	0.6173	0.05

**Table 2:** Inverse correlation of fibronectin and the percentage of cytosines.

	N	df	Pearson’s Correlation	*p*<
All Sgcg–/– mice	28	26	−0.67	0.01
Sgcg–/–Nuc^M^Mito^%M^ mice	17	15	−0.718	0.01
Sgcg–/–Nuc^D^Mito^%M^ mice	11	9	−0.643	0.05

**Table 3:** Primers (IDT, Coralville, IA, USA).

Primer	Sequence
M07 T3900C	5′ TTT CTT TAC CAA TTT TTA CAG GGG GG 3′
M29 T3900C	5′ GGG GAT AAT TGC TAG TAG GCT GAA TA 3′
M30 A tract at 9821	5′ GCT CTT CTA CTT CCA CTA CCA TGA GC 3′
M31 A tract at 9821	5′ GCT ATG GAG CTT ATG GAG TT GAG TT 3′

## Data Availability

The data will be made available by the corresponding author upon reasonable request.

## Supplementary Materials

The following supporting information can be downloaded at: https://www.mdpi.com/article/10.3390/muscles2010005/s1, Figure S1: Neither MRL genome affected muscular dystrophy mediated membrane permeability, as assessed by Evans blue dye uptake; in none of the four tissues analyzed did the MRL genomes affect membrane permeability, N > 10, averages with standard deviations. Figure S2: The adenine length mutation did not segregate with pathology in any of the tissues, by either method of counting the adenine length; shown is the first method used for adenine quantification—just counting the number of visible adenine peaks in each animal.
