# Supplementary material for: The MRL Mitochondrial Genome Decreases Murine Muscular Dystrophy Severity
Source: Muscles. 2023 Jan 16;2(1):0. doi: 10.3390/muscles2010005 (PMC12225360; doi:10.3390/muscles2010005)
Supplement: Supplementary file 1 [file muscles-02-00005-s001.zip › muscles-2132705-supplymentary.pdf]

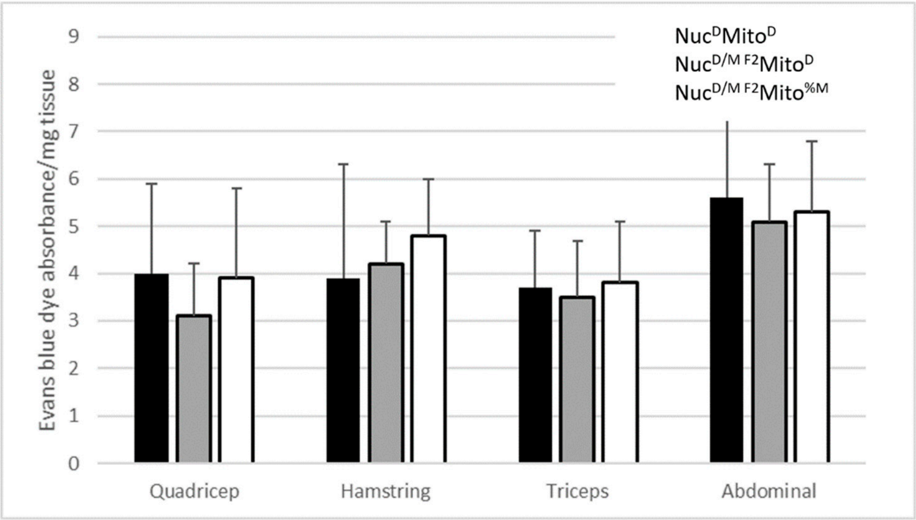

Figure S1. Neither MRL genome affected muscular dystrophy mediated membrane permeability, as assessed by Evans blue dye uptake. In none of the four tissues analyzed did the MRL genomes affect membrane permeability. N>10, averages with standard deviations.

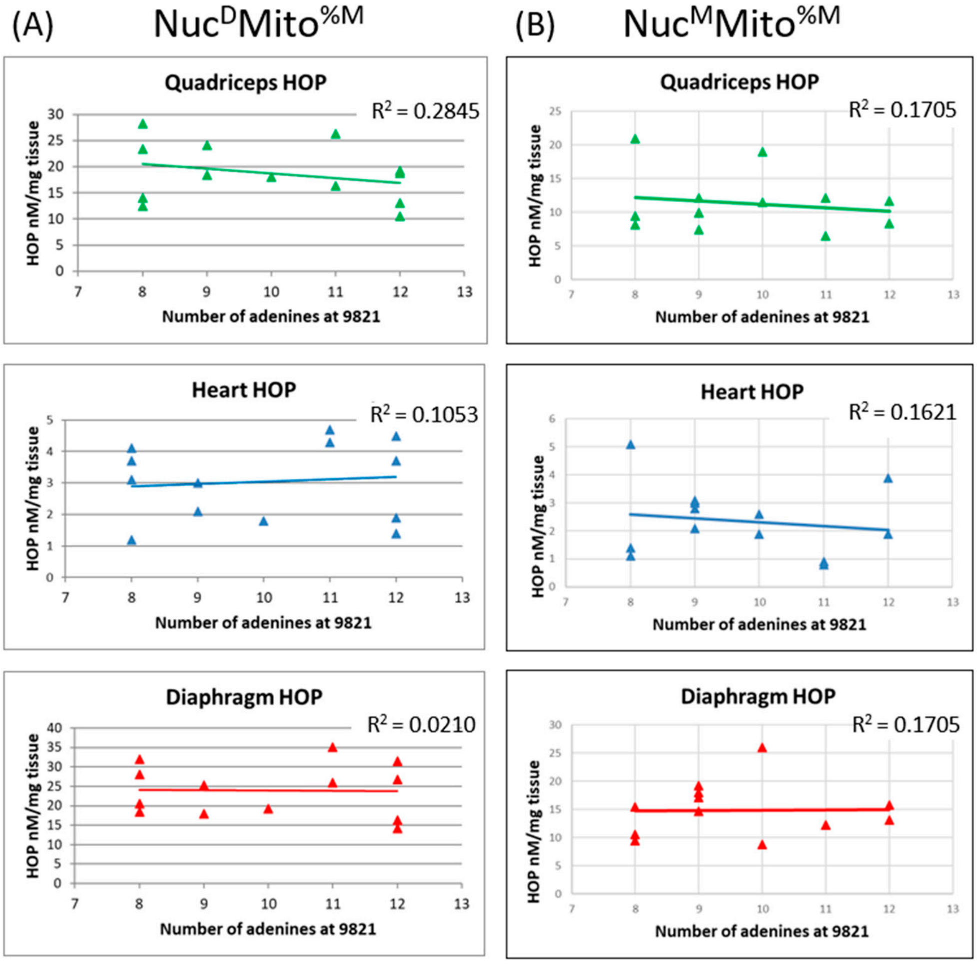

Figure S2. The adenine length mutation did not segregate with pathology in any of the tissues, by either method of counting the adenine length. Shown is the first method used for adenine quantification - just counting the number of visible adenine peaks in each animal.
